# A Community Care Model of Intravenous Antibiotic Therapy for Injection Drug Users with Deep Tissue Infection for “Reduce Leaving Against Medical Advice”

**DOI:** 10.1007/s11469-014-9511-4

**Published:** 2014-08-06

**Authors:** Siavash Jafari, Ronald Joe, Danielle Elliot, Ashnoor Nagji, Sargent Hayden, David C. Marsh

**Affiliations:** 1 Addiction Medicine Program, Vancouver Coastal Health Authority, Vancouver, BC Canada; 2 Department of Social Sciences, York University, 4700 Keele Street, Toronto, ON M3J 1P3 Canada; 3 Community Engagement, Northern Ontario School of Medicine, 935 Ramsey Lake Road, Sudbury, ON P3E 2C6 Canada

**Keywords:** LAMA, Leaving against medical advice, Drug users, Antibiotic therapy, Deep tissue infection

## Abstract

Deep tissue infection is a serious sequela that often demands intravenous (IV) antibiotic treatment. With respect to IV drug users (IDU’s), research and lived experience demonstrates a trend of failed treatment outcomes, most notably associated with leaving hospital against medical advice (LAMA) prior to treatment completion, increased adverse outcomes and patient hardship. This paper examines an alternative model for delivering and completing IV antibiotic treatment to IDU’s in a community care setting. A retrospective study was designed to review client characteristics. A total of 33 in-depth interviews were conducted with clients, clinicians and with staff. The impact of treatment adherence and completion, as well as client satisfaction of care was explored. A total of 165 patients were admitted during the study period. Osteomyelitis was the primary cause for IV antibiotics. Risk of leaving AMA was significantly lower for community model (p value <0.0000). Qualitative narrative analysis is also described with respect to satisfaction, stigma and the need for better models of care. With lower rates of LAMA a community model ought to be considered on a wider scale for provision of comprehensive support for populations with complex underlying health needs.

Patients who are admitted to hospital because of deep tissue infections undergo a course of intravenous (IV) antibiotic therapy until the infection is resolved. Length of treatment varies from a couple of weeks to several months depending on the infection site and severity. After the patients are stabilized within the first days to a week after admission, they often are kept in hospital mainly because of lack of other alternatives in the community to complete the course of IV antibiotic. This results in unnecessary and lengthy hospital stay. Such a lengthy hospital stay not only reduces the efficiency of hospital beds and resources utilization, but also reduces the chance of completion of IV antibiotic therapy through increased risk of leaving against medical advice (LAMA) (Alebiosu and Raimi 2001). We believe a community-based model has the potential to improve completion of planned treatment, increases adherence to care and reduce the length of stay (LOS) in hospitals by providing a patient centered care model. This model not only shares knowledge with clients but involves them in their care planning, takes a team approach, is sensitive to non-medical dimensions of care, and takes into account a harm reduction approach to their substance use behaviour.

In 2005, Vancouver Coastal Health Authority in conjunction with Providence Health Services Authority (PHSA) planned on initiating the “Community Transitional Care Team” (CTCT). CTCT is a community model of care established in response to both increased LOS concerns and the associated mounting acute care bed pressures. This is an innovative and harm reduction intervention in Vancouver’s Downtown Eastside (DTES) offering medical and integrative support in a home-like residence for clients who require long term antibiotic therapy. Acute care clients, in particular those with intravenous drug use (IDU) risks, can have limited access to general home-based intravenous (IV) programs. Subsequently, incurring, a ‘log-jam’ effect in acute sectors for this subgroup often occurs. Emphasizing health holistically, the CTCT is a home-like alternative to hospital to complete IV antibiotics in a safe, medically and socially supported environment. The CTCT accommodates up to nine residents in independent rooms complete with private bathroom, shower, cable TV, telephone, kitchenette, fridge, dining room and mechanically adjustable beds. Staff includes 24 h RN coverage, 24 h Mental Health Workers, daily Physician sessions Monday to Friday with on call 24/7 access, substance dependence counseling and individual case management. Essential clinical care includes, medication management, wound care, lab work and IV antibiotic therapy. Staff accompaniment to all client appointments is mandatory and may include support for appointments with specialists, labs, x-rays, CT and MRI, physiotherapy, occupational therapy, banking, and addictions support meetings. All meals and nutritional requirements are provided. The key program goals are: 1) To increase retention and antibiotic adherence leading to treatment completion; 2) To reduce Leave Against Medical Advice (LAMA); 3) To decrease the average hospital LOS; 4) To facilitate the transition from acute inpatient care to community; 5) To reduce revolving door ER use.

Given the paucity of publications on the effectiveness of community care model on completion of IV antibiotic therapy and risk factors of discharge LAMA among IDU admitted to hospital with soft tissue infection, this mixed-method study seeks to review the profile of and impact on clients admitted to CTCT.

## Methods

In this study we aimed at evaluating the impact of CTCT on completion of antibiotic therapy, LAMA, length of stay, clients’ connection to care and the satisfaction with care among all clients who were admitted to CTCT from hospital. Institutional ethics approval was obtained in advance of the study from the University of British Columbia and related hospitals’ review ethics board. A retrospective data base study was designed to review the characteristics of clients who were admitted to hospital and CTCT between January 1st 2005 and December 30, 2009 with deep tissue infections. We reviewed the profile of all clients who had been admitted to CTCT in this period and collected information on age, gender, diagnosis at admission, underlying medical and psychiatric illnesses, substance use behaviours, homelessness, current medication use, seropositivity for HIV, HCV, and HBV, site of infection, completion rates of antibiotic therapies, LOS at hospital and at CTCT. Data were entered to a Microsoft Access database and then exported to an excel worksheet to allow quantitative analyses. To ensure the accuracy of the data collected, spreadsheets were visually controlled for any missing data and outlier values and inconsistencies were resolved by reviewing the related medical records. Pearson’s chi square test was used to compare the categorical data.

In-depth interviews were conducted with a sub-sample of clients to assess the impact of CTCT on clients’ satisfaction with care. Triangulation was done by interviewing health care professionals from hospital and CTCT to acquire their views about challenges facing providing care to these clients. A total of 33 interviews were conducted with both clients and with hospital and CTCT staff including clinicians (and some administrative personnel) involved in providing care to these clients.

Qualitative data were gathered through in-depth semi-structured interviews consisting of open-ended questions. Each in-depth interview took 1–2 h to complete. All in-depth interviews were audio taped. Data were also collected through direct-observation at the CTCT site where daily interactions, relationships, and settings were observed and recorded through field notes (at various times of the day, on various days of the week to capture the ebb and flow of everyday clinical life). They were encouraged to reflect on clinical interactions, their willingness to engage in biomedical therapies and other programs at CTCT.

In-depth interviews also were conducted with health service providers including staff, clinicians, administrators, and advisory board members, to solicit professional insights and perspectives into compliance, anti-microbial therapy, addiction treatment, and health service delivery for marginalized populations. Interviews were also sought from other professional experts in the field of acute medicine delivery, infectious disease, and emergency medicine. Narrative analysis used to explore the themes and subthemes. All interviews were transcribed and the field notes were added to the appropriate areas of each transcript.

## Results

A total of 165 patients, 94 (57 %) male and 71 female (43 %) were admitted to CTCT during the study period. Mean and standard deviation (SD) of age for all participants was 41 (9.3) years with a range of 16 to 77 years old. Female clients on average were 2.7 years older than males. One hundred and thirty nine of clients (84 %) were using at least one illicit drug at admission and of them 65 (39 %) were injection drug user. Opioids (65 %) crack/cocaine (58 %), marijuana (14 %), alcohol (14 %) and crystal methamphetamine (10 %) were the most common drugs of dependence among these clients. Also, 44 % of clients were using both opioids and crack/cocaine whereas ongoing use opioid or crack/cocaine with alcohol was reported by 11 and 10 % of clients respectively.

Although our clients were from a relatively young age group (mean age at admission = 42 years), they had several underlying medical conditions at admission. Table 1, represents the prevalence of underlying medical illnesses among participants. As seen hepatitis C infection (64 %) was the most common underlying illness amongst participants, followed by diagnosis of at least one mental illness (46 %) and HIV (34 %).

**Table 1:** frequency of the underlying illnesses among clients

Illness category	N (165)	Percent
Hepatitis C	107	64 %
Mental Illness	74	46 %
HIV	57	34 %
Endocarditis	31	19 %
Anemia	27	16 %
Asthma	20	12 %
Hepatitis B	13	8 %
Heart Failure	11	7 %
Hypothyroid	8	5 %
High blood pressure	9	5 %
Diabetes mellitus	7	4 %
COPD	7	4 %
Other cancers	6	4 %
Renal failure	4	2 %
Cirrhosis	3	2 %
AIDS	3	2 %
Liver cancer	2	1 %

Note: Some participants had multiple underlying illnesses so the sum is over 100 %

Our results also indicate that 15 (10 %) clients had one medical condition, 27 (17 %) clients had two medical conditions, 46 (29 %) clients had three medical conditions, 27 (17 %) clients had four medical conditions, 22 (14 %) clients had five medical conditions, 12 (8 %) clients had six medical conditions, 3 (2 %) clients had seven medical conditions, 4 (3 %) clients had eight medical conditions, and 1 clients (1 %) had nine medical conditions.

As explained before, clients are admitted to CTCT for continuation of their IV antibiotic therapy. Osteomyelitis was identified as the most common reason for initiation of IV antibiotics among 51 (31 %) clients, followed by abscess among 22 cases (13 %), septic arthritis among 17 cases (10 %) and cellulitis among 14 cases (8 %). Infection of the lumbar vertebra was reported among 22 cases (13 %) and was the most common site of infection. Table 2 represents the most common sites of infection among clients admitted to CTCT.

**Table 2:** Type of infection at admission and infection site

Infection site	N (115)	Percent
Lumbar spine	22	19 %
Foot	19	17 %
Leg	13	11 %
Sacroiliac	12	10 %
Hip joint	12	10 %
Arm/Forearm	11	10 %
Shoulder	10	9 %
Cervical spine	6	5 %
Knee	4	3 %
Ankle	4	3 %
Thigh	2	2 %

**Fig. 1:**
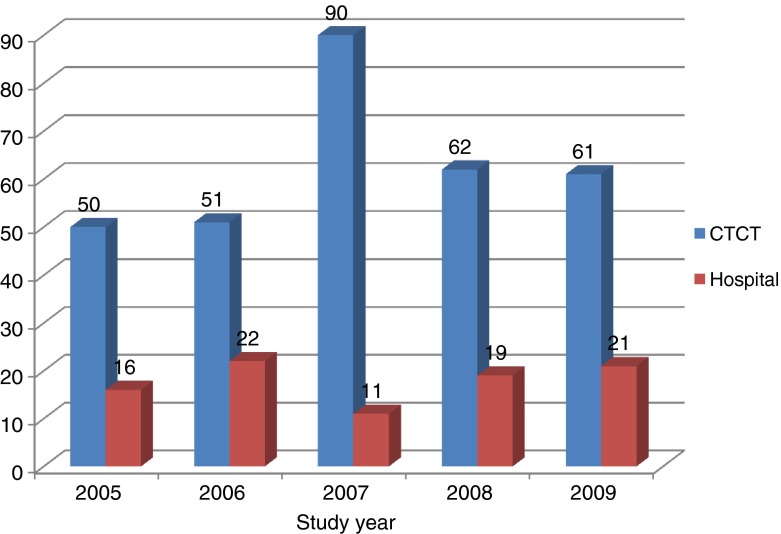
Length of stay in hospital and CTCT

Figure 1 represents the LOS of clients in hospital compared to CTCT. As seen, the LOS in hospital was between 16 and 22 days in the study period. However, for the same clients the LOS at CTCT ranged from 50 to 90 days.

Our findings also indicate that the risk of leaving AMA was significantly lower among clients who were staying at CTCT. In fact, out of 50 AMAs that occurred in this period, only two AMAs occurred among clients who were staying at CTCT, in contrast to the 48 AMAs which occurred amongst hospital cases (P Value < 0.0000).

**Fig. 2:**
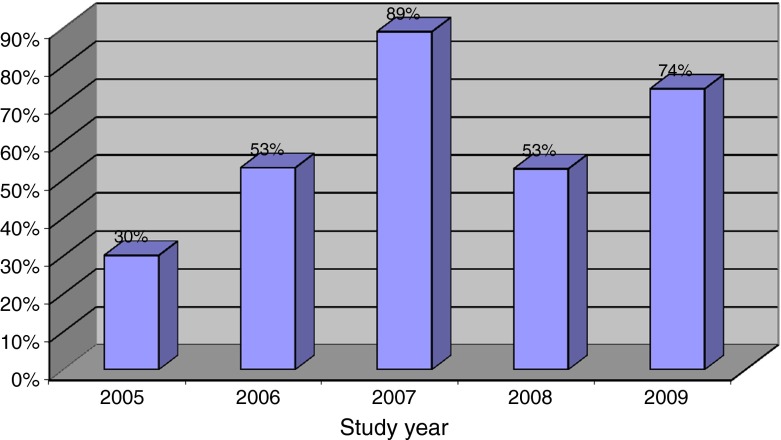
Percent of total capcacity used in each study year

The number of bed days filled at CTCT for each year was calculated. As shown in Fig. 2, the CTCT was using 30 % of its available capacity in the first year, and this steadily increased to 74 % in 2009. The highest bed-day filled occurred in 2007 which may have been due to two cases who had long LOS.

Another important accomplishment of CTCT is discharging clients to a stable housing. A total of 59 (36 %) of clients were homeless in admission and all of them were discharged to a stable housing at the completion of their antibiotic therapy.

### Qualitative Results

In-depth interviews were conducted with 8 CTCT clients and 25 health service providers. These interviews were supplemented with field observations and notes based on dozens of informal conversational-like discussions with key informants including developers and managers of the CTCT. The following themes were discovered to be consistently reported by both clients and care providers.

### Satisfaction

One hundred percent of the participants favoured receiving better care and treatment at the CTCT compared to any of the hospital sites that they had experienced. They reported that the CTCT program is welcoming, sensitive to their social and medical needs, and characterized the staff as skilled and understanding. The following quotations are indicative of the favorable assessments by respondents:

They accept you for who you are. You don’t have to be anybody else. You can be yourself there. Um, they have good staffing there. I get along with all the staff, and they help you out if you can’t do something. I didn’t expect to be catered to the way I was but I clearly enjoyed it. (F1)

My room is nice. I have fresh air. Better than you have in the hospital Male One. …I don’t like (the) hospital. I don’t like the way they treat you or anything. … They’re really bad. (The) CTCT literally saved my life. (F2)

### History of Leaving Care AMA

The respondents reported that their histories of leaving acute care against medical advice (AMA) were often due to negative social and psychological responses by staff in the acute setting, as opposed to an unwillingness to receive care.

I don’t like the hospital. I don’t like the way they treat (you) when you’re a junkie, like they don’t say that but the way their attitude is. I’ve seen them treat other people, oh, so nice and kind…. but if you’re from the Downtown Eastside then, you’re garbage. …I think I walked out [AMA], because I just had enough of it.

A couple of times that I was in there [hospital] I had nurses that just drove me insane. They bitched me out and then, on the other hand I had fantastic nurses that I just loved, you know.

This was further supported by other health-care providers working in the Downtown Eastside as the following anecdote indicates:

… part of the issue was that she wasn’t being heard [at the hospital] and, the other issue was that it was welfare day. And they were supposed to help her go get her cheque and she had this deal with herself that she was only gonna use on welfare day, right? So, she had a whole lot of anxiety about getting out there and getting it over with basically. And they were not helping her. They had to do something to the dressing. And she was waiting and waiting, …

### Stigma and Moral Judgments

In particular, participant narratives suggest that there continues to be a lack of comprehension of the complexity of drug addictions by some staff, moral judgments in nursing and clinical care, and an intense stigmatization of homeless, impoverished patients – especially if they engage in drug use within the acute care setting.

And, last week, um, 2 weeks ago I got sick and last week I ended up in the hospital. And uh, when I went there, the head nurse said, “Oh my God! You’re back!” You know, and that didn’t feel good…. They kept accusing me of smoking in my room. They even accused me… I’d been in, in the operating room, all day once, right? To get this PICC in, you know, it took them 3 h to get it in. Okay, so I had been under anaesthetic that whole time, … screaming in pain, even under anaesthetic, but when I came out of the anaesthetic, they said they could smell alcohol. [sigh] And I didn’t even drink at the best of times. You know, but I insisted that they take a blood test and stuff. And I was glad they did because, [pause] it cleared it right up.

Most health-care providers interviewed disagreed with the participants’ suggestion that they were treated poorly in the hospitals while, concomitantly, acknowledging that patients with chronic injection drug use habits tend to be deemed “difficult”, “non-compliant” and “chaotic.” The CTCT staff for the most part embraced a different philosophy of care – emphasizing health holistically (as defined by psycho-social, political, economic, and biological forces) instead of a biomedical framework emphasizing physical or bodily health. This was represented in everyday health-care practices:

… But it’s about building a trusting relationship. A non-judgmental, meeting people where they’re at. And, you know, having that compassionate, acceptance of, the kind of challenges that people are having in their lives…. if you’re working in a place like that, you know, you need to have a level of compassion that accepts that if somebody’s coming from … people have their own histories and are what they are for legitimate reasons…

### Need for a Better Care Model for this Clients

All health-care providers and administrators agreed that there was a definite need for developing alternative models of care and treatment for individuals engaging in chronic and acute injection drug use who are in need of long-term antibiotic treatment.

… We’re treating people the same … we’re doing the same thing for a completely different patient population. It’s not working. We’ve got to do something different.

… we’ve been pushing for it for 5 years now, to get some, some other, you know, alternative better place to treat patients that don’t really want to be in the hospital…

… It’s an inappropriate venue, I guess I would say for treating them cause I don’t think the hospital is necessarily, for most of them, is the appropriate venue for treatment, or for treating them.

… Who knows what the outcome, but, certainly the hospital didn’t do well. Not because the people weren’t well intentioned… But in the end it was just the wrong place for this guy. And, we failed him. And he failed us…We know they do so poorly in hospital…

… People with mental illness and chronic addictions have been seen as being broken and, in a number of ways deficient. In our system, forever it’s been seen as appropriate and okay, to either deny them treatment, or provide them substandard treatments… and the reason why this been okay is that the perception of the population are broken and deficient - and not the system. The perception of the system is that the system’s fine and, it’s their fault that they can’t stay in hospital. Or it’s their fault that they can’t complete treatment. These people aren’t broken, you know they’re, not deficient. They’ve got conditions that require treatment. Until those conditions are treated, it’s our obligation as health care providers, to provide them [with] services that suit their needs, and is accessible to them. And that’s what this is all about. It’s about providing service, it’s saying that the system was broken.

### Dealing with Difficult Patients

Health-care providers working in the hospitals reported on-going frustration in patient management due to various reasons including cost effectiveness, staff safety, patient care and drug use.

… As a [health-care] provider certainly I’m sometimes in the position of threats, intimidation, verbal abuse at times. But I think other people experience it even more than I do. You know, sexual innuendos and touching that is inappropriate, inappropriate language, swearing and, objects being thrown, so it’s challenging work with the population…

… I think the really big issue is around behavioral issues, from the drug use. Sometimes people can go really off the deep end and difficult behaviors frighten other people, the staff uh, unsafe uh, like really uh, unsafe practices. We’ve had patients lighting fires in the building. They cook up their drugs and, burning bed sheets and so on…

There was a general consensus that, due to a myriad of complex reasons, the primary-care setting continued to fail the most vulnerable of patients and that the CTCT project could address that on-going challenge. Respondents expressed their hopes that other components, like a supportive recovery arm will be implemented soon. Combined, the different components are understood to provide the basis for a more comprehensive model of care that is patient-centered and addresses the spectrum of needs posed by the range of addiction and medical issues being presented in these patients.

## Discussion

In this study we assessed the impact of a community transition care model on completion of antibiotic therapy among individuals with deep tissue infections. Our findings indicate that a community transition care model can be a very successful model in providing care to clients with complex needs. CTCT is providing care to a group of clients who have active infection and require IV antibiotic therapy but are likely to leave the hospital AMA. These clients have complex health conditions, are on multiple medications, and are likely to be poly substance users and homeless, all of which may increase the LOS at hospital, while they can be managed in community where basic resources are provided in a home-care setting. The benefits of community care have been well documented and now serve as best practice (Pope 2003). Given the effectiveness of an integrated approach to health care, many authors have written about potential advantages of a multidisciplinary model to respond to intertwined diseases of addictions, mental health and other co-infections (Taylor 2005).

At various teaching and acute care hospitals in the United States, leaving AMA accounts for 0.8–2.2 % of discharges (Weingart, Davis, and Phillips 1998; Smith and Telles 1991; Jeremiah, O’Sullivan, and Stein 1995; Anis et al. 2002). Furthermore, significant predictors for poorer outcomes and higher rates of rehospitalization (Hwang, Li, Gupta, Chien, and Martin 2003) have been found in those leaving AMA and in substance users. In particular, among injection drug users (IDU), recurrence of skin and soft tissue bacterial infections are frequent (Saitz et al. 2000; Gibbs, Hamill, and Magruder-Habib 1991 and are often complicated by need for surgical intervention, repeat hospital visits, poor adherence to antibiotic regimens and high rates of AMA (Crane, Levine, Zervos, and Cummings 1986; Levine, Crane, and Zervos 1986; Binswanger et al. 2000; Center for Disease Control 2001). In this study we found that 48 AMAs occurred at hospital whereas only 2 AMAs occurred at CTCT for the same clients in the same time period. Our community care model improves the adherence to care and completion of IV antibiotic therapy. The benefits of such accomplishment are several folds. This model of care not only facilitates clients’ connectedness to care, but also it promotes completion of antibiotic therapy which improves outcomes for the patient while reducing the risk of antibiotic resistance. Poor adherence to care and completion of antibiotic therapy could result in development of severe and invasive infection such as sepsis and transmission of infection within the community. Successful treatment of infections would also reduce the hospital re-admission rates.

A recent review of literature found that structured discharge plan tailored to the individual patient probably brings about small reductions in hospital length of stay and readmission rates so the impact of discharge planning on mortality, health outcomes and cost remains uncertain (Shepperd et al. 2010). Discharging clients who are homeless and have no place to go is however a challenging task. It is very common to see that a patient is discharged from the medical service however is unable to leave the hospital because of lack of a stable housing. This extended use of hospital beds is considered one of the seven wastes of hospital resources described by NHS (NHS 2008). CTCT plays an important role on admitting such clients from hospital and reducing the burden on health care system and on clients. As shown, 36 % (59) of clients who were admitted to CTCT were homeless and had no place to be discharged to. It is common also that some clients to be discharged from hospital to shelters after staying long enough to complete their treatment course when no stable housing is manageable at discharge. A pre-discharge planning at CTCT facilitates provision of a stable housing and in fact no client is discharged unless a stable housing is ensured. This would result in reduction in homelessness within the community which have significant clinical and public health implications such as increased all-cause mortality, difficulty accessing primary care, increased emergency room visits, and poor chronic disease control.

Our community transition care model contributes to a significant reduction on the LOS in the hospital which has several downstream benefits. Studies have found that increased LOS in hospital is associated with increased mortality even after adjustment for severity of illness (Flood, Ewy, Scott, Forrest, and Brown 1979). Another benefit of reduction of LOS would be reduction of average cost per stay in hospital. In 2009, $52.9 billion of Canada’s annual Canada’s 182.1 billion health care fund was used by hospitals, which accounts to 29.1 % of total annual health care budget (CIHI 2011). Reducing LOS will release capacity in the system, including beds and staff time which could result in reduction in wait time to accessing the care.

Another important finding of this program is the heightened access by female clients. In other studies, women have been found to be susceptible to marginalization that results in underutilization of care (Brady and Randall 1999). Such a high access and connectedness to care creates an opportunity to engage female substance users in addiction and HIV care and treatment which would result in lower risk of transmission of blood borne infections such as HIV, HCV, HVB, through provision of treatment and injection drug use prevention (Puentes-Markides 1992).

We believe that there are several key factors that are central to the success of the CTCT model. First is the home-like setting –a private space that mimics a safe and comfortable home-like environment. This provides the clients a sense of control in their daily life while normalizing essential clinical care and medical oversight. Second, staff embrace an intimate understanding of the resident ‘life-world’ social backdrop thus engendering a culture of acceptance and participation. Third, psychosocial support and quality ‘face time’ with residents builds relationships and enhances engagement opportunities between the client and caregiver. Finally, wholesome home-made meals are tailored to resident requests further maximizing participation and nutritional uptake.

With lower rates of AMA, higher adherence to and completion of antibiotic therapy, improved access to housing, and provision of care to clients with complex underlying health needs we believe this innovative community-based care model ought to be considered on a wider scale for comprehensive, patient-centered medical and integrated support for at-risk populations.
